# Astaxanthin Bioactivity Is Determined by Stereoisomer Composition and Extraction Method

**DOI:** 10.3390/nu14071522

**Published:** 2022-04-06

**Authors:** Terry W. Snell, John Carberry

**Affiliations:** 1 School of Biological Sciences, Georgia Institute of Technology, Atlanta, GA 30332, USA; 2 Sustainable Aquatics, 110 W. Old Andrew Johnson Highway, Jefferson City, TN 37760, USA; johnc@mosseycreekenterprises.com

**Keywords:** astaxanthin, stereoisomers, micronutrient, bioavailability, bioactivity, mitochondrial targeting, metabolic effects

## Abstract

Astaxanthin (ASX) is a natural product and one of the most powerful antioxidants known. It has significant effects on the metabolism of many animals, increasing fecundity, egg yolk volume, growth rates, immune responses, and disease resistance. A large part of the bioactivity of ASX is due to its targeting of mitochondria, where it inserts itself into cell membranes. Here, ASX stabilizes membranes and acts as a powerful antioxidant, protecting mitochondria from damage by reactive oxygen species (ROS). ROS are ubiquitous by-products of energy metabolism that must be tightly regulated by cells, lest they bind to and inactivate proteins, DNA and RNA, lipids, and signaling molecules. Most animals cannot synthesize ASX, so they need to acquire it in their diet. ASX is easily thermally denatured during extraction, and its high hydrophobicity limits its bioavailability. Our focus in this review is to contrast the bioactivity of different ASX stereoisomers and how extraction methods can denature ASX, compromising its bioavailability and bioactivity. We discuss the commercial sources of astaxanthin, structure of stereoisomers, relative bioavailability and bioactivity of ASX stereoisomers, mechanisms of ASX bioactivity, evolution of carotenoids, and why mitochondrial targeting makes ASX such an effective antioxidant.

## 1. Introduction

Astaxanthin is a xanthophyll carotenoid molecule, a natural product of algae, yeast, and marine invertebrates such as shrimp and krill, and can be synthesized from petroleum. It is one of the most powerful antioxidants in nature and has significant effects on the metabolism of many animals, increasing fecundity, egg yolk volume, growth rates, immune responses, and disease resistance. Recently it has been recognized as a mitochondrial-targeting antioxidant (MTA). Consequently, this molecule has been the subject of extensive research for the past 20 years.

Metabolically active astaxanthin (ASX) (3S,3′S) is a natural product from the green alga *Haematococcus pluvialis* and is an esterified 3,3′-dihydroxy-β,β-carotene-4,4′-dione, with a molecular formula of C_40_H_52_O_4_ and a molar mass 596.84 g/mol. It has 40 carbon atoms organized into two oxygenated iononetype rings, joined by a polyene chain [1,2]. The conjugated double bond chain acts as a strong antioxidant by electron donation and by reacting with free radicals [3] and is responsible for its deep red color. Hydroxyl acid on terminal rings can react with fatty acids and can form mono- or diesters. ASX is commonly found conjugated with proteins in salmon muscle and in lobster exoskeleton. Interacting with fatty acids or proteins stabilizes ASX, which is unstable in its free form and particularly susceptible to oxidation. Free ASX is the form produced synthetically from petroleum distillation and produced by yeast [3]. ASX is naturally found as stereoisomers in free and esterified forms. ASX can assume different conformations, including chiral (3S,3′S) or (3R,3′R) or meso forms (3R,3′S), with the chiral stereoisomers most prevalent. In addition, the polyene chain double bonds can assume cis or trans conformations, but the trans isomer is most common due to instability of the cis isomer.

Astaxanthin is an important micronutrient for many animals and humans, with potential pharmacological effects, including anti-diabetic, anti-inflammatory, and antioxidant activities, as well as cardiovascular, ocular, and skin-protective effects [4]. This is because ASX acts as a powerful antioxidant protecting mitochondria and other sensitive cellular organelles from damage by reactive oxygen species (ROS). ROS are ubiquitous by-products of energy metabolism that must be tightly regulated by cells, lest they bind to and inactivate proteins, DNA and RNA, lipids, and signaling molecules, especially in the mitochondria, which are in close proximity to the source of ROS. Mitochondria are the source of a large amount of cellular ROS; as much as 2–3% of all the oxygen processed by mitochondria is lost as ROS [5]. Most animals cannot synthesize ASX, so they need to acquire it in their diet. ASX is an example of an important micronutrient that has been greatly reduced in animal and human diets by the industrialization of agriculture and food processing [6]. ASX is easily thermally denatured during extraction, and its high hydrophobicity makes it difficult to maintain high bioavailability.

The purpose of this review is to contrast the bioactivity of different ASX stereoisomers and how extraction methods can denature ASX, compromising its bioavailability and bioactivity. For these reasons, it is important for investigators to specify the source and stereoisomer of ASX used in their studies. Failure to do so can lead to the lack of reproducibility of work among labs, an underestimation of ASX effects, and an excessive number of false negative results. We describe a source and extraction method that produces the metabolically active 3S,3′S stereoisomers that are esterified and micelle/liposome encapsulated. This form of ASX has the greatest bioavailability and bioactivity for a variety of animal and human applications. The 3S,3′S esterified stereoisomer has a unique and powerful tendency to take up residence in lipid bilayers of cell membranes, where it powerfully inactivates excess ROS without interfering with the signaling roles of ROS [7].

## 2. Materials and Methods

The analyzed literature consists of journal articles from the last two years in Google Scholar retrieved using astaxanthin as key word. The search returned >13,000 articles. We sorted through these articles seeking to understand the bioavailability and bioactivity of this molecule in relationship to its stereoisomer structure, subcellular binding partners, metabolic activity, and antioxidant activity. The analysis was performed between late 2021 and early 2022.

## 3. Results

### 3.1. Sources of Astaxanthin

Astaxanthin today is sourced from a petroleum distillate, from the yeast *Phaffia rhodozyma*, from the green alga *Haematococcus pluvialis*, or by extraction from the shells and biomass of marine arthropods such as shrimp and krill [8]. These sources of ASX provide a mixture of the three different stereoisomers. Astaxanthin possesses two identical asymmetric atoms at C-3 and C-3′ making possible three optical isomers with all-trans configuration of the chain: 3S,3′S, 3R,3′S, and 3R,3′R (Table 1). The distribution of the isomers in natural ASX differs from that of the synthetic product. This latter is a racemic mixture, with a typical ratio of 1:2:1 (3S,3′S:3R,3′S:3R,3′R), while ASX from natural sources is typically 3S,3′S [9]. The angles on the C-3 and C-3′ favor this isomer taking up residence in the cell plasma membrane since these are angled to anchor the hydrophilic elements (the rings) of the ASX in the hydrophilic elements of cell membranes (the lipid “heads”), and the hydrophobic element of the ASX (the conjugated backbone) with the hydrophobic elements of cell membranes (the lipid “tails”). 

The main source of the 3S,3′S stereoisomer is the green alga *H. pluvialis*, which is common in small, ephemeral freshwater bodies that are widely distributed globally. It is well suited for survival under harsh conditions of extreme light, temperature, and salt concentration that would be lethal to many other microalgae. *H. pluvialis* is able to tolerate these extreme environments because of its ability to rapidly switch from a green growth phase to a red encysted phase that can tolerate desiccation [17]. The mature encysted cells accumulate large amounts of secondary carotenoids, particularly ASX, in lipid droplets deposited in the cytoplasm, resulting in a bright red color. Encysted cells can remain metabolically dormant for many years, as long as environmental conditions remain hostile (hot, dry, saline). Once environmental conditions turn favorable again, encysted cells germinate into green flagellated cells and initiate a new vegetative growth cycle.

When stressed, the photosynthetic machinery of *H. pluvialis* cells is reorganized and ASX is accumulated. Oxidative stress and ROS accumulation are triggers for activation of the ASX biosynthetic pathway. This includes β-carotene overproduction in the chloroplast followed by export to the cytosol and conversion to ASX. The key enzyme, β-carotene oxygenase (CRTO), produces ASX from β-carotene or zeaxanthin, is found in plastids, lipid vesicles, and in the cytosol [18]. Astaxanthin rich oil droplets accumulate in the cytoplasm and probably have a role in protecting the nucleus and other cellular organelles from oxidative and UV damage.

Accumulation of ASX is the key adaptation that allows *H. pluvialis* encysted red cells to tolerate desiccation and other extreme environments. To resist the oxidative stress of these conditions, the total carotenoid content of encysted red cells becomes 80–99% ASX. The majority of ASX is deposited within the cell as fatty acid esters of ASX, usually mono- or diesters of palmitic (16:0), oleic (18:1), or linoleic (18:2) acids [12]. Under certain stressful environmental conditions, *H. pluvialis* accumulates up to 3–5% of its dry weight as ASX, primarily the bioactive 3S,3′S stereoisomer.

**Figure 1:**
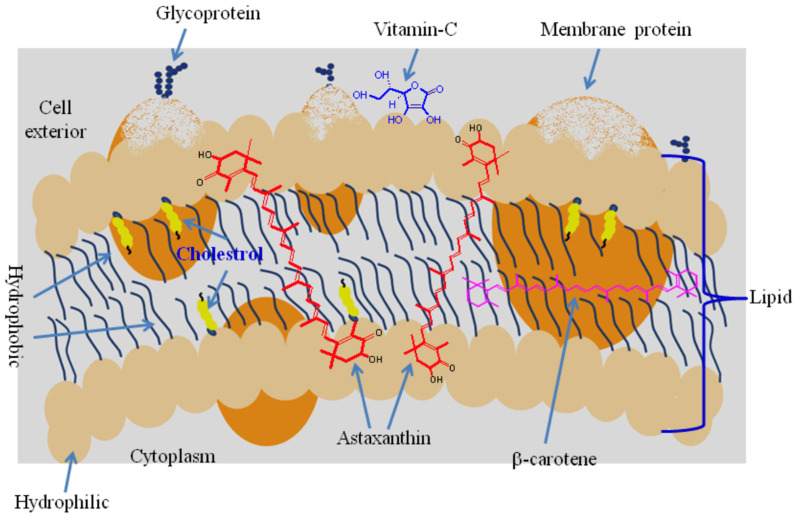
Position of astaxanthin in the cell membrane bilayer. (From: Ambati et al. [2].)

Although the main industrial source of natural ASX today is from the *H. pluvialis*, it is not bioavailable in its extracted form [19]. The most popular method for extracting ASX is super-critical carbon dioxide extraction, which denatures the molecule and does not make it bioavailable. Using a laser microtrac, we measured the *H. pluvialis* cyst at 60 µm and the product produced by super-critical carbon dioxide extraction to be 4–6 µm, which is still biologically unavailable. The challenge of extracting ASX from *H. pluvialis* is to do so at low temperatures to avoid denaturing the molecule [20]. A U.S. patent teaches a method for leveraging the rich protein and lipid and carotenoid content of the *H. pluvialis* by using a high-shear, low-temperature process with an abundance of food-grade ethanol to reduce the biomass to less than 100 nm. With this method, upon low-temperature, low-pressure removal of the ethanol, the ATX is esterified and encased in micelles and liposomes, which protect the ester groups and makes the molecule highly bioavailable [21]. This allows complex assembly of structures around bi-esterified ASX within micelle and liposome structures. In this conformation, the 3S,3′S angles of the esterified oxygen and hydroxyls on the rings are oriented towards the heads of the lipids in the cell membrane bilayer, where they can be very bioactive as antioxidants (Figure 1). In contrast, synthetically produced ASX is composed of stereoisomers that are angled awkwardly, so they are unable to fit into the cell membranes and consequently float free in cells. Here, ASX binds to ROS, interfering with the ROS signaling function, which is why synthetic ASX is so toxic to young fish.

The key to making ASX efficacious as an antioxidant is to make it bioavailable. This is done by constructing micelle and liposome structures around the ASX molecule. The cell membrane is a bilayer of lipids, with heads facing out and tails facing in (Figure 1). The esterified 3S,3′S ASX stereoisomer inserts itself into the bilayer where it is stable and bioavailable for antioxidant activity.

The thickness of phospholipid bilayers is about 5 nm and the length of the esterified ASX is about 6 nm. Its molecular structure is configured so that it takes up residence in the bilayer and reinforces the strength and resilience of this otherwise fragile structure [22]. From this site, it also is well positioned to absorb excess ROS generated by energy production in the mitochondria, without interfering with ROS signaling functions. This insertion into the membrane bilayer also explains why the color in salmon or trout flesh bleeds out when placed into ice and water for storage. These fish are typically fed synthetic or yeast-produced ASX, which are mainly 3R,3′R isomers that float freely in animal cells without membrane insertion. In contrast, the 3S,3′S isomers of *H. pluvialis* firmly insert into the cell membrane.

The stability and bioavailability of 14 ASX esters with different molecular structures were investigated using in vitro and in vivo digestion models [20]. They demonstrated that ASX esters with long-chain and saturated fatty acids were more stable than other types of ASX esters. Astaxanthin diester was more stable than astaxanthin monoester and free ASX. The results indicated that ASX esters with short-chain fatty acids had higher bioavailability than ASX esters with long-chain fatty acids, whereas ASX esters with highly unsaturated fatty acids had higher bioavailability than ASX esters with low unsaturation fatty acids. Lin et al. [23] performed a bioinformatic analysis on ASX binding affinity for a variety of molecular targets. They identified its affinity targets and potential pharmacological activity by perturbing signaling pathways.

### 3.2. Differential Activity of Astaxanthin Stereoisomers

The different ASX stereoisomers have differential bioactivities because of their different antioxidant capacity, different interactions with metabolic signaling pathways, different localization patterns within cells, and affinity for different types of free radicals. The key metabolic effects in animals are increased fecundity, growth rate, egg yolk volume and quantity, intensity of flesh color, and strengthening of immune responses.

We have confirmed the bioactivity of the *H. pluvialis* esterfied 3S,3′S isomer, encapsulated in micelles and liposomes over several years in the course of our work on fish nutrition. The Sustainable Aquatics hatchery is one of the largest marine ornamental fish hatcheries in the world. For the last 20 years, it has produced more than 200 species of fish in recirculating aquaculture systems (RAS) on a diet that includes ASX. This has enabled the hatchery to avoid the use of antibiotics or vaccines that are typically required in most fish hatcheries. In addition, they have observed 99% yield eggs to sale with virtually no sickness. They also have near complete success in managing breeding pairs, some with decades of twice-a-month spawning, observing superior color, few deformities, and fast growth. When ASX was included in the diets of Sustainable Aquatics larval clownfish, they grew faster, matured faster, and achieved darker coloration than controls lacking ASX. Astaxanthin dietary supplementation increased in clownfish nest counts by 2/3 and produced 2.1 times faster weight gain by clownfish larvae [24]. Similarly, ASX supplementation improved salmon growth and flesh color in RAS culture systems [25].

The *H. pluvialis* esterified 3S,3′S isomer also has strong bioactivity in rotifers, live foods critical for rearing marine fish larvae. For example, supplementation of the rotifer *Brachionus manjavacas* with ASX yielded up to 43% faster reproductive rates, 46% higher population densities, and more stable mass cultures. As a powerful antioxidant, ASX also markedly enhanced rotifer resistance to oxidative stress, a common cause of collapse in rotifer mass cultures, allowing rotifer populations to reach and sustain higher densities [26].

The stereoisomer-specific bioactivity of ASX makes it imperative that researchers describe the source and extraction method for the ASX that they use in their experiments. We expect very different results from ASX prepared synthetically from petroleum or extracted from yeast. These stereoisomers are primarily 3R,3′R, which have substantially lower bioactivity than the 3S,3′S isomers from *H. pluvialis*. It is therefore not appropriate to compare bioactivities of different stereoisomers because doing so could lead to false negative results. It is also important to state the extraction method used to obtain the ASX. When ASX is extracted using the super-critical CO_2_ method, ASX is typically exposed to high temperatures, which denatures the molecule. Clearly, bioassays with this material will lead to much lower estimates of activity than undenatured ASX. Consequently, if the full potential of ASX as a dietary supplement in animal and human nutrition is going to be accurately determined, the stereoisomer tested and the extraction method must be specified.

### 3.3. Mechanisms of Astaxanthin Bioactivity

At molecular levels, ASX prevents oxidative damage through various mechanisms, including quenching singlet oxygen, scavenging radicals, inhibiting lipid peroxidation, and regulating oxidative stress-related gene expression [27]. ASX’s antioxidant activity is largely attributed to its interactions with cell membrane lipids [28]. In contrast to other carotenoids, the polar structure of ASX allows it to be incorporated into cell membranes. This decreases lipid peroxidation and does not produce harmful pro-oxidative effects [29]. Furthermore, ASX inhibition of lipid peroxidation is related to its ability to trap ROS within and on both sides of the membrane [30]. Growing evidence suggests that ASX improves mitochondrial function by reducing the impact of mitochondrial ROS, increasing ATP production, and increasing mitochondrial number and respiratory chain complex activity [31,32].

### 3.4. Evolution of Carotenoids

Xanthophyll carotenoids probably evolved from as early as 3.8 billion years ago in photosynthetic bacteria and archaea and then with the earliest eukaryotic cells about 2.5 billion years ago [33]. Carotenoids were essential to protect biomolecules from the combustion products of energy metabolism. The molecular pathways of photosynthesis require a variety of cofactors, including several carotenoids. Coevolution of carotenoids with the biomolecules of photosynthesis is one of the key reasons for the remarkable efficiency of photosynthesis in contemporary autotrophs [34]. Respiration consumes oxygen and produces waste products, including carbon dioxide and a considerable amount of free radical oxygen. These include oxygen singlets and hydrogen peroxide, H_2_O_2_. About 2–3% of the oxygen processed in eukaryote cells becomes ROS [5]. The most prominent of these is ROS, unavoidable by products of the metabolic reactions in chloroplasts and mitochondria.

**Figure 2:**
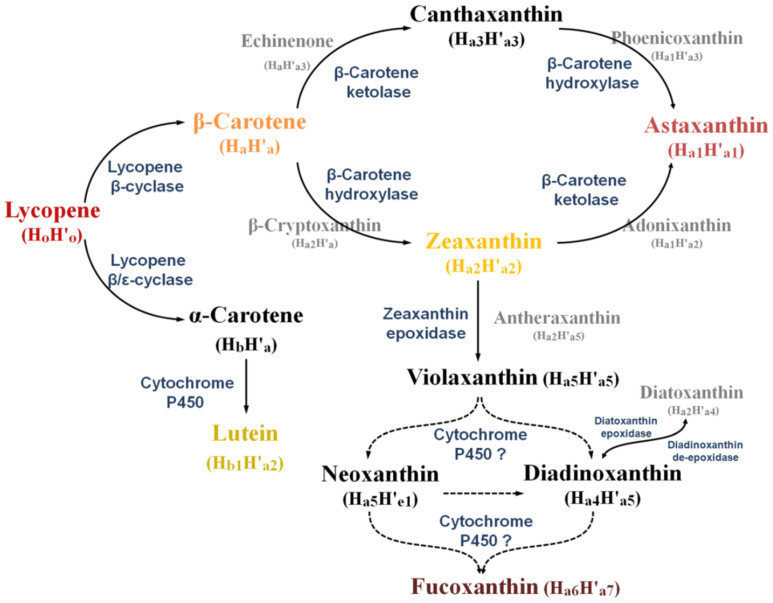
Metabolic pathways for astaxanthin synthesis from lycopene. Arrows indicate catalyses, with enzymes shown in blue. Dashed arrows indicate hypothesized reactions. Reaction intermediates are shown in gray. (From: Wang et al. [35].)

As cells became more active, they evolved higher production of adenosine triphosphates (ATP) to support their metabolism. With the evolution of multicellularity, this increased demand for biochemical energy grew more intense. Natural selection favored cells capable of protecting their biomolecules from the damaging ROS co-generated by energy production. A variety of carotenoid antioxidants evolved, including lycopene, b-carotene, lutein, canthaxanthin, zeaxanthin, and astaxanthin (Figure 2).

Xanthophyll carotenoids are a class of oxygen-containing molecules that create the color of many of the yellow, orange, and red hues in flowers, fruits, vegetables, egg yolks, feathers, shells, and flesh of many animal species (flamingo, canary, shrimp, lobster, chicken, or salmonids). In plants, they are involved in photosynthesis with chlorophyll and are responsible for the red, yellow, and brown colors of autumn foliage as chlorophyll is degraded. Compared to other xanthophyll carotenoids, which have 11 double bonds, ASX is the most reactive molecule in this family, with 13 double bonds. Other than some arthropods, most animals do not make ASX, so it is an essential dietary micronutrient. In addition, to be bioavailable, it must be incorporated into micelles and liposomes so that it can be absorbed from the intestinal epithelium into the blood by endocytosis.

## 4. Discussion

### 4.1. Mitochondrial Targeting Makes Astaxanthin an Exceptionally Effective Antioxidant

Although oxygen is an essential electron acceptor for energy production, it is very dangerous in cells as a free radical or singlet atom because it is so reactive. Therefore, it must be tightly controlled, especially in mitochondria where reactive oxygen leakage can destroy normal metabolism. To gain a sense of this problem, at rest, the average human body uses 1 kg of oxygen per day. During maximal exercise this increases to 10 to 20 g per minute. One to two percent of this processed oxygen is lost in typical mitochondrial processing, and this translates to 10 to 20 g O_2_ lost per day at rest and 200 mg per minute at maximal exercise. The amount of mass and energy processed by a healthy person at rest equals their body weight in adenosine triphosphate every day.

Mitochondria can take up as much as 25% of the cell volume, with average cells containing from 400 to 2500 mitochondria. They are sites of high metabolic activity, constantly producing ATP to meet the energy demands of cells. Mitochondrial metabolism represents a huge source of oxidative stress for cells. The primary way mitochondria are protected from this intense oxidative stress is the deployment of antioxidant enzymes and molecules to scavenge oxygen free radicals. Examples of endogenous antioxidant enzymes include superoxide dismutase (SOD) and catalase [36,37]. These work in tandem with nonenzymatic antioxidants, such as glutathione, and dietary antioxidants, such as vitamin E and ASX [38]. Different antioxidants target different parts of the cell, quenching different types of free radicals. Consequently, a suite of both endogenous and dietary antioxidants is needed to maintain proper redox homeostasis in cells.

Ferro et al. [39] have shown that dietary supplements of ASX support mitochondrial function, protecting its redox balance. Astaxanthin significantly reduced oxidative stress and kept mitochondria in a reduced state, even after exposure to H_2_O_2_ [30]. Astaxanthin also prevented the loss of the mitochondrial membrane potential and the escape of electrons with increased consumption of oxygen by mitochondria [40]. Astaxanthin can prevent mitochondrial dysfunction by permeating and co-localizing within the mitochondria [41]. Astaxanthin protects mitochondria-rich muscles during exercise because it modulates redox homeostasis and limits exercise-induced inflammation [42]. Astaxanthin also co-localizes with mitochondria because they are abundant in cells, they are rich in membranes, and astaxanthin is very hydrophobic, being most stable when it is inserted into the bilayer of a membrane.

As a free radical scavenger, ASX is more than 65 times stronger than vitamin C and 50 times more powerful than vitamin E in protecting cell membranes [43]. In addition, ASX has been shown to be more effective than other carotenoids at singlet oxygen quenching by being up to 800 times stronger than coenzyme Q, 6000 times greater than vitamin C, 550 times more powerful than green tea catechins, and 11 times stronger than b-carotene. It is also a 2.8 times stronger antioxidant than lutein. These antioxidant properties of ASX illustrate why it is often beneficial in treatment of cardiovascular, immune, inflammatory, and neurodegenerative diseases [44,45].

### 4.2. Mitochondrial Dysfunction Is at the Core of Many Contemporary Diseases

Mitochondria are the organelles producing the most adenosine 5′-triphosphate (ATP) and ROS in eukaryotic cells, and consequently are susceptible to oxidative damage. Irreversible oxidative damage in mitochondria has been implicated in many human diseases [46]. There is increasing evidence that mitochondria-targeted antioxidants such as ASX have considerable potential for treating oxidative damage-associated diseases [47].

An example of a disease at least partially caused by mitochondrial dysfunction is diabetes mellitus (DM). DM results from high blood sugar and is associated with oxidative stress and inflammation [48]. Astaxanthin has been shown to be beneficial therapy in diabetic retinopathy and neuropathy by activating the NF-κB pathway, suppressing microvascular injuries by VEGF generation, and anti-apoptotic activity via modulation of MAPKs and PI3K/Akt pathways [49]. Furthermore, ASX inhibits NF-κB translocation, transforming growth factor-beta (TGF-β) generation, inflammation, and fibrosis. Astaxanthin also has been shown effective in treating cardiovascular complications associated with diabetes, secondary to anti-inflammation and anti-oxidation modulation via the NF-κB pathway [32].

Studies such as these are accumulating in the scientific literature, and we expect mitochondria-targeted antioxidants such as ASX will play an increasingly prominent role in treating chronic diseases such as DM.

## 5. Conclusions

The most metabolically active form of ASX is a lipid-rich, nano-emulsified, esterified molecule of the 3S,3′S stereoisomer, which is made bioavailable by encapsulating in micelles and liposomes. Proper extraction of ASX from the green alga *Haematococcus pluvialis* provides this form of ASX, in contrast to other natural products from yeast or synthetic products from petroleum. In this conformation, the 3S,3′S angles of the esterified oxygen and hydroxyls on the rings are oriented towards the heads of the lipids in cell membrane bilayers where they can insert and become very bioactive as antioxidants. We have confirmed the bioactivity of the *H. pluvialis* esterified 3S,3′S isomer, encapsulated in micelles and liposomes over several years in the course of our work on fish nutrition. Dietary supplements of ASX have been reported as effective in treating several human diseases.

## Figures and Tables

**Table 1:** Characteristics of astaxanthin stereoisomers.

Stereoisomer	Primary Source	Localization	Bioavailability	Bioactivity
3S,3′S 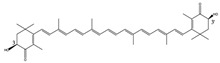	*Haematococcus pluvialis* [10] *Paracoccus carotinifaciens* [11] *Salmo salar* [2]	Mitochondria, membrane insertion	high	Anti-oxidation Anti-inflammatory Facilitates mitochondria function Strengthens immunity Promotes fertility UV protection Colorant
3R,3′S 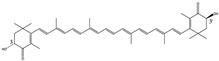	*Penaeus monodon* [12] *Litopenaeus vannamei* [13] Synthetic—petroleum [14]	Float freely in cells	low	Anti-oxidation Colorant
3R,3′R 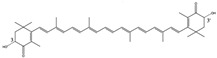	*Phaffia rhodozyma* [15] *Euphausia superba* [16]	Float freely in cells	low	Anti-oxidation Colorant
